# Computer Vision Analysis for Objective Motor Assessment in Parkinson's Disease: A Retrospective Study

**DOI:** 10.1002/mdc3.70488

**Published:** 2025-12-20

**Authors:** Pasquale Maria Pecoraro, Luca Marsili, Antonio Cannavacciuolo, Kevin R. Duque, Jesus Abanto, Jennifer Sharma, Jennifer Scheler, Heba A. Deraz, Lauren Wingler, Vincenzo Di Lazzaro, Alberto J. Espay, Lazzaro di Biase, Matteo Bologna

**Affiliations:** ^1^ Operative Research Unit of Neurology, Fondazione Policlinico Universitario Campus Bio‐Medico Rome Italy; ^2^ Research Unit of Neurology, Neurophysiology and Neurobiology, Department of Medicine and Surgery, Università Campus Bio‐Medico di Roma Rome Italy; ^3^ James J. and Joan A. Gardner Center for Parkinson's Disease and Movement Disorders, Department of Neurology, University of Cincinnati Cincinnati OH USA; ^4^ IRCCS Neuromed Pozzilli Italy; ^5^ Department of Neurology Queens University Kingston ON Canada; ^6^ Department of Radiology Nuclear Medicine, University of Cincinnati Cincinnati OH USA; ^7^ Neurology department Cairo University Hospitals Cairo Egypt; ^8^ Brain Innovations Lab Università Campus Bio‐Medico di Roma Rome Italy; ^9^ Department of Human Neurosciences Sapienza University of Rome Rome Italy

**Keywords:** bradykinesia, computer vision, digital biomarkers, machine learning, quantitative analysis, telemedicine

## Abstract

**Background:**

The Movement Disorder Society‐Unified Parkinson's Disease Rating Scale‐Part III (MDS‐UPDRS‐III) is subjective and insensitive to subtle changes in patients with Parkinson's disease (PD). Computer vision (CV) can extract objective kinematics from routine outpatient videos, potentially augmenting the accuracy of the motor assessment.

**Objective:**

We set out to (1) Identify CV‐derived finger‐tapping features that discriminate PD from healthy controls (HC); and (2) Quantify the relationship of these extracted features with clinical and Dopamine Transporter Single‐photon Emission Computed Tomography (DAT‐SPECT) anchors in PD patients.

**Methods:**

We retrospectively analyzed outpatient finger‐tapping videos from PD patients with DAT‐SPECT positivity within one year from videos and HC. A Mediapipe‐based pipeline quantified tapping velocity, changes in amplitude, and variability in amplitude and rhythm. Diagnostic performance was estimated with Receiver Operating Characteristic Area Under the Curves (ROC AUC) and 95% Confidence Interval (CI). Spearman assessed the relationship between tapping features, MDS‐UPDRS‐III, item 3.4 (finger tapping), and disease duration.

**Results:**

Thirty‐two PD patients and ten controls were included. Amplitude variability (AUCs, 0.93; 95% CI, 0.84–0.99, *P* < 0.001), and rhythm variability (AUC, 0.83; 95% CI, 0.69–0.94, *P* < 0.001) exhibited the best discriminatory capacity for a PD diagnosis. MDS‐UPDRS‐III correlated positively with amplitude variability (ρ = 0.55, *p* = 0.001) and amplitude decrement (ρ = 0.414, *P* = 0.009), and negatively with tapping velocity (ρ = −0.34, *P* = 0.05). Amplitude variability (ρ = 0.387, *P* = 0.014), and rhythm variability (ρ = 0.304, *P* = 0.045) directly correlated with item 3.4, while amplitude variability positively correlated with disease duration (ρ = 0.39, *P* = 0.026).

**Conclusion:**

CV‐derived tapping variability features objectively discriminated PD from healthy subjects and tracked motor severity.

Despite refinements of current diagnostic criteria, the clinical diagnosis of Parkinson's disease (PD) is still suboptimal and suffers from an error rate of ~20%.^1^, ^2^, ^3^, ^4^, ^5^, ^6^, ^7^, ^8^ Traditionally, evaluation of motor performances relies on examiner‐rated scales such as the Movement Disorder Society–Unified Parkinson's Disease Rating Scale (MDS‐UPDRS) and Hoehn & Yahr staging. While invaluable, these tools are limited by subjectivity, inter‐rater variability, and reduced sensitivity to subtle longitudinal change. As a result, they may fail to capture clinically meaningful details.^9^, ^10^, ^11^, ^12^, ^13^

This shortfall underscores the need for validated objective biomarkers to characterize motor performance. To this end, wearable sensors^14^, ^15^, ^16^, ^17^ and Internet of Things (IoT) architectures^18^ are being explored to augment detection of PD motor symptoms, while markerless radio‐frequency monitoring^19^ and Artificial Intelligence (AI)‐based approaches^20^, ^21^ further support continuous, and scalable assessment. Computer Vision (CV) can quantify kinematic aspects of movement directly from routine videos, providing objective, scalable, and fine‐grained measurements of motor performance. It is particularly attractive since it requires no body‐worn hardware, leverages commodity cameras, and extracts large amount of data with high temporal resolution in naturalistic settings.^22^, ^23^, ^24^ Indeed, it takes advantage of an essential component in the toolbox of the movement‐disorder expert: videorecording.^25^

Beyond simply mirroring clinical ratings, recent literature has supported the potential of CV to generate new insights into the relationship between motor performance and clinical/demographic variables.^26^ Linking quantified motor behavior to patient characteristics such as age, sex, disease duration, or severity may help clarifying the heterogeneity of PD. In this regard, analyzing the finger tapping with DeepLabCut, a CV‐derived score demonstrated high accuracy in distinguishing PD patients from healthy controls (HC) (91% sensitivity, 97% specificity), significantly aligning with clinical rating scales.^27^ A MediaPipe‐based framework analyzed finger‐tapping videos from PD and HC to extract kinematics from the thumb–index angle, achieving an Area Under the Curve (AUC) of 0.97. Amplitude and speed declined linearly with severity, whereas variability‐related features were nonlinear.^28^

Moreover, CV could enable scalable, reproducible phenotyping across large populations and diverse clinical and research settings. While the MDS‐UPDRS‐III remains the clinical gold standard for motor assessment in PD, its ratings are gross ordinal scales which may miss subtle cycle‐to‐cycle kinematic changes during repetitive movements. By extracting detailed temporal and spatial movement features, CV enables a more precise characterization of motor impairment and may reveal dimensions of disease not captured by conventional scales.^27^, ^29^ In this regard, a recent large‐scale work in PD quantified >1200 clinical video recordings and, using sparse principal component analysis, identified three levodopa‐responsive kinematic domains of finger tapping—speed, consistency, and movement timing/scale—unveiling robust objective, reproducible motor endpoints beyond clinician‐rated scales.^29^

Despite rapid progress in pose‐estimation methods, few studies have delivered a clinic‐ready pipeline that (i) relies on a single, ≤15‐s task with the most‐affected hand, (ii) yields interpretable features aligned with MDS‐UPDRS‐III item 3.4 (finger tapping), and (iii) is anchored to an imaging reference standard to mitigate diagnostic misclassification. Moreover, the relationship between finger‐tapping kinematic metrics and established clinical anchors (MDS‐UPDRS‐III total and item 3.4), disease duration, and DAT‐SPECT striatal binding ratio (SBR) remains incompletely characterized in routine outpatient conditions.

To address this need, we applied a custom CV pipeline to retrospectively analyze finger‐tapping outpatient videos from a well‐characterized cohort of PD patients, all with clinical diagnoses confirmed by Dopamine Transporter Single‐photon Emission Computed Tomography (DAT‐SPECT) imaging, as well as HC. In this study, we set out to explore the feasibility of applying retrospectively the CV technology, with the following aims: (1) To assess whether CV‐derived finger‐tapping features are sensitive markers to the PD diagnosis; (2) To quantify associations between these CV‐derived finger‐tapping features and key demographic, clinical, DAT‐SPECT anchors in PD patients. Overall, this approach may provide a valuable framework for both clinical practice and research, by enabling objective, data‐driven characterization of motor performance in PD.

## Methods

### Eligibility Criteria

Patients referred to the outpatient clinic of University of Cincinnati Gardner Neuroscience Institute (UCGNI), with a clinical diagnosis of PD according to Movement Disorders Society (MDS) diagnostic criteria^1^ were retrospectively evaluated. Inclusion criteria were: (1) Available standardized videos of bilateral finger tapping collected during regular outpatient neurological examinations; (2) Videos recorded with a smartphone at a fixed frame rate of 30 frames per second (fps), resolution ≥720p, landscape orientation, and a fixed camera on a stable surface. Uniform lighting and a neutral background were required. The tapping hand had to remain continuously in focus and fully visible without occlusions, for 15 s of uninterrupted tapping (as previously outlined by our group^24^); (3) Confirmation at quantitative assessment of nigrostriatal dopaminergic denervation on DAT‐SPECT imaging within one year (±1 year) from collected videos.

Two neurologists (LM, PMP) extracted the following data from electronic medical records: sex, age at videorecording, age at motor symptoms onset, Montreal Cognitive Assessment (MoCA) score collected within one year from the video, disease duration, total MDS‐UPDRS‐III score, Hoehn & Yahr stage, Levodopa Equivalent Daily Dose (LEDD) (mg/day, as per Tomlinson and colleagues^30^), and DAT‐SPECT information related to regional SBR. All patients were videotaped under their regular dopaminergic therapy, namely in the practically defined ON state,^31^ within 2 hours of the last levodopa dose or at the time participants reported having reached their usual ON state.

In addition, HC performing finger tapping with the dominant hand were consecutively enrolled and videotaped. HC were defined based on the following criteria: (1) Normal neurological examination; and (2) No previous exposure to anti‐dopaminergic drugs. Subjects unable to perform the task due to orthopedic/osteoarticular limitations (painful range‐of‐motion restriction, recent upper‐limb injury) were excluded.

### DAT‐SPECT Acquisition Protocol

In order to minimize thyroid gland uptake, 500 mg of potassium iodide oral solution (SSKI) were administered to patients before intravenous injection of 185 MBq of 123I‐FP‐CIT. DAT‐SPECT imaging was performed 3 h after the radiopharmaceutical injection using a dual‐head gamma camera (Infinia Hawkeye, GE 630, GE 670, GE ECAM, GE Healthcare, Milwaukee, WI). A semiquantitative evaluation of the striatal distribution of 123I‐FP‐CIT was done through DaTQUANT™ 2.0 Software (GE Healthcare, Milwaukee, WI). The software enabled fully automated registration of SPECT images onto a 3D‐volume model of interest derived from a large database of healthy scans and were age‐matched to the patients. Regional radiotracer uptake was quantified with the Striatal Binding Ratio (SBR), expressed as: $\mathrm{SBR}=\frac{\mathrm{SBR}\;\mathrm{ROI}-\mathrm{SBR}\;\text{background}}{\mathrm{SBR}\;\text{background}}$. Mean ROI uptake is the average tracer uptake in the striatum, putamen, and caudate nucleus, while the mean background count is the average tracer uptake in the occipital cortex with negligible DAT expression, representing nonspecific uptake of the tracer.

### Computer Vision Analysis

The finger‐tapping task was standardized to 15 s for all participants and recorded under uniform conditions: participants were seated in an armchair and videos were acquired against a white, uncluttered background. Acquisition used a single fixed camera on a stable surface in the frontal (anterior) plane. Finger tapping was analyzed from the most affected hand in PD patients, and from the dominant one in HC, consistently with our previous studies.^32^, ^33^, ^34^, ^35^

**Figure 1:**
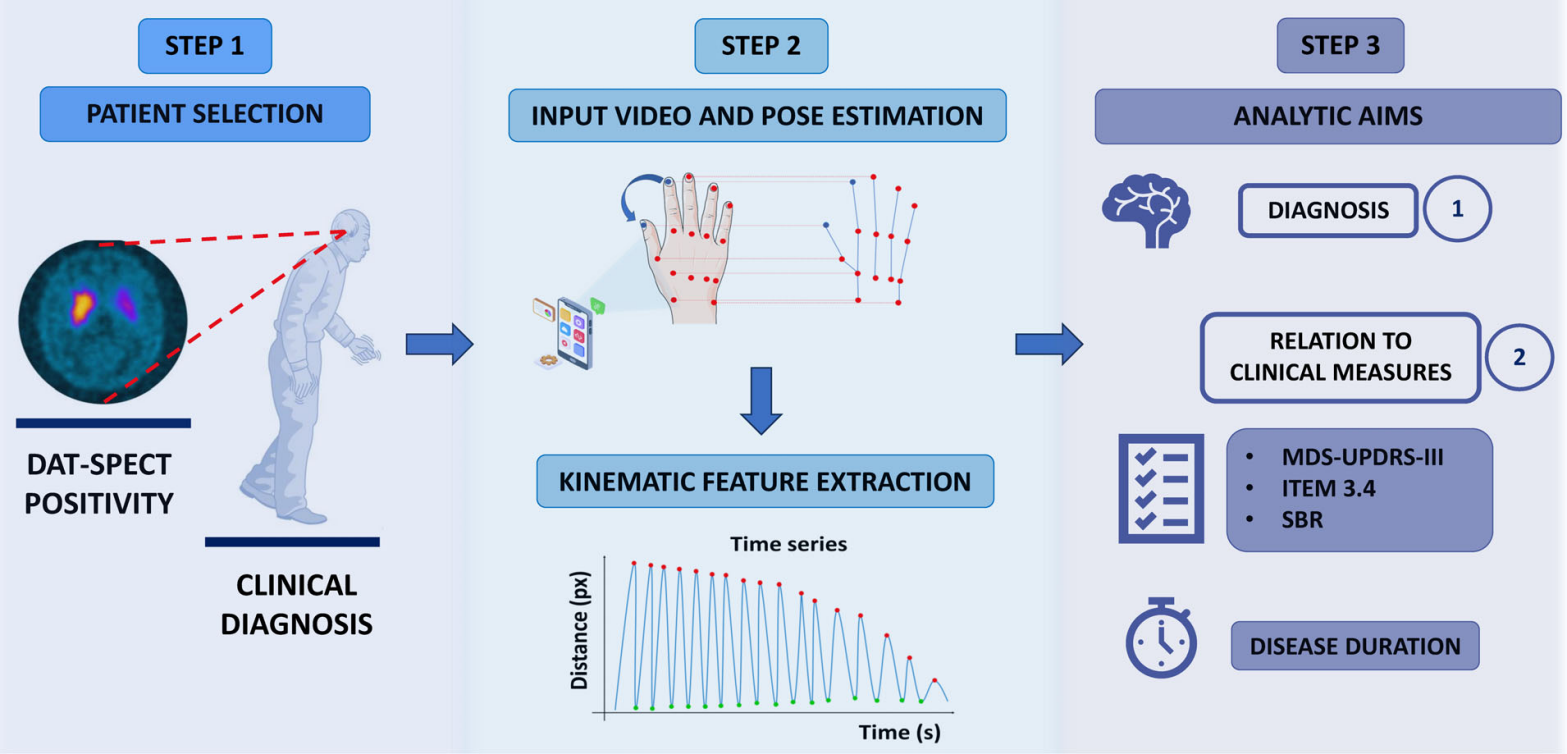
Pipeline of the study. Patients were selected by clinical diagnosis of PD and DAT‐SPECT positivity, then underwent outpatient smartphone finger‐tapping recording. Videos were processed with markerless pose estimation to track index–thumb landmarks, yielding a distance‐over‐time signal from which kinematic features aligned with MDS‐UPDRS III item 3.4 were extracted (velocity, amplitude decrement, amplitude variability, rhythm variability). Analyses addressed for diagnostic classification (PD vs controls) using these features, and their relations to clinical anchors (MDS‐UPDRS‐III total and item 3.4), DAT‐SPECT SBR, and disease duration. DAT‐SPECT, Dopamine Transporter Single‐photon Emission Computed Tomography; MDS‐UPDRS‐III, Movement Disorders Society‐Unified Parkinson's Disease Rating Scale; SBR, striatal binding ratio.

All videos were processed with a custom CV software derived from Mediapipe (*Mediapipe*, Google Inc, Version 0.10.26, Jul, 10, 2025), an open‐source software of Google (Google Inc, Mountain View, CA, USA) for automated markerless pose estimation and landmark detection. Preprocessing included linear‐trend removal on the distance trace, 2nd‐order Savitzky–Golay smoothing (window defined a priori by frame rate and expected tap cycle), and peak detection with refractory interval to avoid double counts. Quality control excluded segments with >5% dropped/blurred frames, hand occlusion, or loss of landmarks; retained segments had ≥15 s of continuous tapping. The CV software predicted pixel (px) coordinates (x, y, image‐normalized) of key points for each video. To evaluate over‐time variations in finger tapping, the tips of the index and thumb fingers were chosen as landmarks for the pose estimation. The software derived a waveform from index–thumb landmarks and quantified finger‐tapping kinematic features over 15 s. To harmonize timing, signals were resampled to 50 Hz when needed and filtered with a 5th‐order Butterworth filter. Hand landmarks were used to compute a frame‐wise index–thumb waveform; from this, we derived finger‐tapping kinematic features aligned with MDS‐UPDRS‐III item 3.4: velocity (V), amplitude decrement (Δa), amplitude coefficient of variation (aCoV, a measure of amplitude variability), and instantaneous frequency coefficient of variation (ifCoV, a measure of rhythm variability). Tapping velocity was expressed in pixels per second (px/s). Δa was the slope of the linear regression expressed in px per cycle, representing the change in movement amplitude across successive repetitions. aCoV was expressed as CoV = σA/μA, where μA represents the mean of the amplitudes in a number of taps, σA is the standard deviation of the amplitudes. ifCoV was defined as ifCoV = σif/μif, where **i** *f* represents the instantaneous frequency calculated for each time interval Δt between consecutive taps (*if* = 1/Δti) and σf is the standard deviation of the instantaneous frequencies (*if*), μf is the mean of the instantaneous frequencies in a number of intervals. Figure 1 summarizes the pipeline of the study.

### Statistical Analysis

Normality of continuous variables was tested using the Shapiro–Wilk test. Group differences in continuous variables were analyzed with parametric or non‐parametric tests as appropriate, while sex distribution was assessed with chi‐square tests. For neuroimaging data, SBR values from DAT‐SPECT were log‐transformed and analyzed with a mixed‐effects repeated‐measures model adjusted for disease duration. This model evaluated differences across striatal ROIs (striatum, caudate, putamen, putamen‐to‐caudate ratio) and between hemispheres. The discriminative ability of finger‐tapping kinematic features was assessed with receiver operating characteristic (ROC) analysis. ROC curves were generated using predicted probabilities from binary classification PD vs HC, and AUC, standard error (SE), 95% confidence intervals (CI), *p*‐values were further calculated. Binary logistic regression was further applied to estimate the odds of correctly classifying PD patients from HC based on finger‐tapping kinematic features‐including Cox–Snell R^2^, Nagelkerke R^2^, and Wald. Spearman's rank correlation examined the relationship between kinematic features and clinical measures, including MDS‐UPDRS‐III total score, item 3.4, and disease duration.

In correlation plots, 95% CIs around the fitted regression line were estimated using non‐parametric bootstrap resampling (1000 iterations). The significance threshold was set at *P* < 0.05. Multiple comparisons were corrected with the Benjamini–Hochberg procedure (false discovery rate, FDR = 5%), and adjusted *q*‐values were reported. All statistical analyses were performed with SPSS version 29.0 (IBM Corp., Armonk, NY). Data visualization and graphical outputs were generated in Python (Python Software Foundation, Wilmington, DE).

## Results

### Flow of Patient Selection

**Figure 2:**
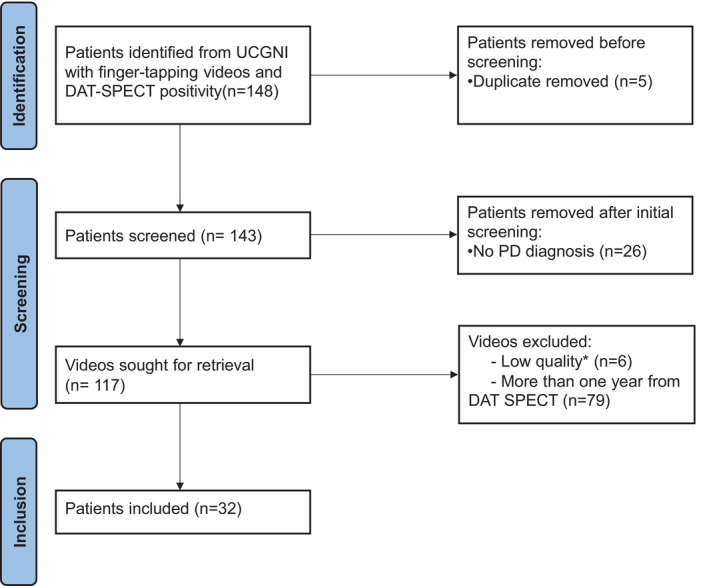
Flow diagram of participant and video selection for the DAT‐SPECT–anchored PD cohort. DAT‐SPECT, Dopamine Transporter Single‐photon Emission Computed Tomography; UCGNI, University of Cincinnati Gardner Neuroscience Institute.

We retrospectively identified 148 candidates with finger‐tapping videos from outpatient clinic and with DAT‐SPECT positivity. After removing duplicates (*N* = 5, 3.4%), 143 unique patients were screened; 26 without a PD diagnosis (atypical parkinsonism) were excluded (18.2%), leaving 117 videos for retrieval. Following quality control, 85 recordings were excluded. 79 videos were excluded because the video was obtained >1 year from DAT‐SPECT positivity (72.6% of those sought). Afterwards, 6 videos were excluded for low quality: variable frame rate, slow‐motion, noticeable motion blur, >5% dropped/corrupted frames, or severe compression artifacts. The final pooled cohort comprised 32 PD patients (21.6% of those identified) (Fig. 2) and 10 HC.

### Demographic and Clinical Characteristics

Pooled cohort was composed of 32 PD patients meeting inclusion criteria within one year of DAT‐SPECT imaging positivity, and 10 age‐ and sex‐matched HC.

Shapiro–Wilk tests showed that Age at onset and Age at video approximated normality (*P* > 0.05). In contrast, MoCA, LEDD, MDS‐UPDRS III, Hoehn &Yahr stage, disease duration, and DAT‐SPECT SBR measures violated normality (*P* < 0.05). Among kinematic tapping features, Δa was normally distributed (*P* > 0.05), whereas V, aCoV, and ifCoV were non‐normal (*P* < 0.05) (Table S1).

Two‐sample Welch's t‐test found no difference in Age at video between the pooled PD cohort and HC (t = 1.253, *P* = 0.218). A Mann–Whitney *U* test did not find any difference in MoCA between groups (U = 190.5, *P* = 0.645). A chi‐square test revealed no significant association between both groups and sex (χ^2^(1) = 0.025, *P* = 0.875). In mixed‐effects models adjusted for disease duration, no significant right–left hemisphere differences in log‐transformed(SBR) were found across ROIs: striatum β = −0.360 [95% CI, −1.070, 0.350], *P* = 0.320; putamen β = 0.440 [95% CI, −0.420, 1.300], *P* = 0.316; caudate β = 0.460 [95% CI, −0.370, 1.290], *P* = 0.277; putamen/caudate ratio β = −0.160 [95% CI, −0.470, 0.140], *P* = 0.296 (Table S2).

Demographic, clinical and DAT‐SPECT features of the study population are presented in Table 1.

**TABLE 1:** Demographic, clinical and DAT‐SPECT features of the study population

Type	Variable	Pooled PD cohort	HC	*P*‐value
Clinical	Participants	32	10	‐
	Sex (male)	60%	50%	*P* > 0.05
	Age at videorecording	60 ± 11	65 ± 2.4	*P* > 0.05
	Age at motor symptoms onset	52.6 ± 13	‐	‐
	MoCA	26 ± 2	27.4 ± 1	*P* > 0.05
	Disease duration (years)	8 ± 10	‐	‐
	Total MDS‐UPDRS‐III	25.3 ± 15	‐	‐
	Hoehn & Yahr stage	1.87 ± 0.7	‐	‐
	LEDD (mg/die)^27^	482 ± 709	‐	‐
DAT‐SPECT	Most affected hand (Contralateral)			‐
	Striatum	1 ± 0.6	‐	
	Caudate	1.33 ± 0.7	‐	
	Putamen	0.9 ± 0.5	‐	
	Putamen‐to‐caudate ratio	0.8 ± 0.2	‐	

Abbreviations: LEDD, Levodopa Equivalent Daily Dose; MDS‐UPDRS, Movement Disorders Society‐Unified Parkinson's Disease Rating Scale; MoCA, Montreal Cognitive Assessment.

### Diagnosis

**Figure 3:**
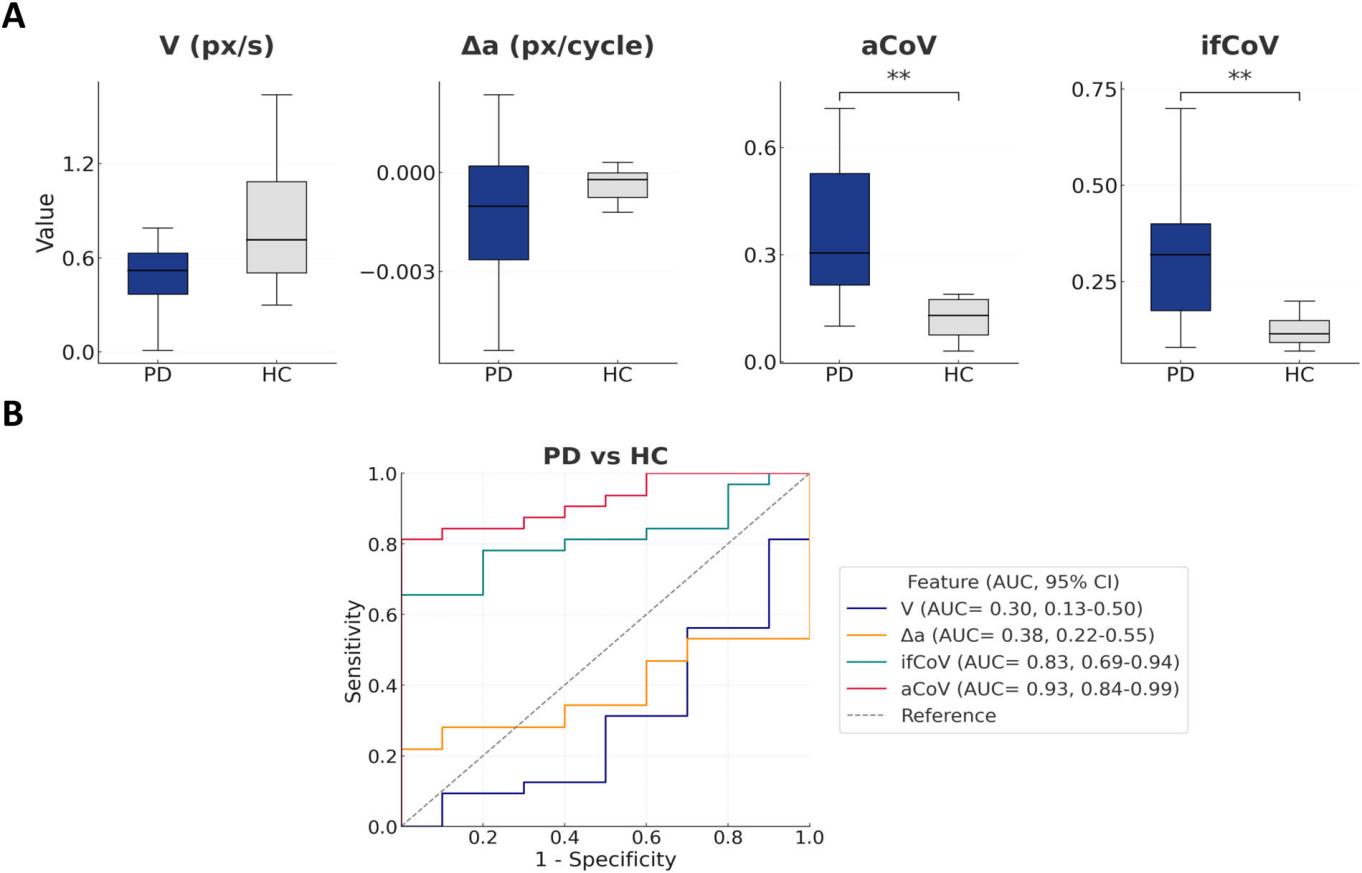
(A) Boxplot distribution of finger‐tapping kinematic features extracted via CV analysis in PD and HC. (B) ROC curve with AUC (95% CI), standard error (SE) and *p*‐values for binary classification of PD vs HC. ** = *P* < 0.01. Δa, amplitude decrement; aCoV, amplitude coefficient of variation; HC, healthy controls; ifCoV, instantaneous frequency coefficient of variation; PD, Parkinson's disease; px, pixels; V, Velocity.

Two‐tailed Mann–Whitney tests comparing PD with HC found that aCoV (U = 297.5, *P* < 0.001), and ifCoV (U = 266, *P* = 0.0018) were significantly higher in PD, indicating large effects. Indeed, tapping V tended to be lower in PD but did not reach significance (U = 97.5, *P* = 0.067), and Δa did not differ between groups (U = 114.0, *P* = 0.179) (Fig. 3, Panel A). After explicit matching by sex and nearest‐age, the results were confirmed. Variability metrics displayed significantly higher values in PD: aCoV 0.33 [0.28–0.56] vs 0.13 [0.075–0.175] (U = 86.5, *P* < 0.001) and ifCoV 0.405 [0.338–0.620] vs 0.115 [0.093–0.150] (U = 96.0, *P* < 0.001). V tended to be lower in PD (0.45 [0.155–0.61] vs 0.715 [0.505–1.085]) with a trend to significance (U = 24.5, *P* = 0.0586), whereas Δa showed no group difference (−0.0007 [−0.0022–0.0001] vs −0.0002 [−0.0008−−0.0000]; U = 39.0, *P* = 0.4274).

ROC analysis showed the following AUC values: aCoV 0.93 (95% CI, 0.845–0.986, SE = 0.037, *P* = 0.0001), and ifCoV 0.83 (95% CI, 0.697–0.936, SE = 0.062, *P* = 0.0018) were the most informative. By contrast, tapping V trended below chance and did not reach significance (AUC 0.31; 95% CI, 0.137–0.500, SE = 0.092, *P* = 0.067), and Δa showed no discriminatory value (AUC 0.36; 95% CI, 0.203–0.519, SE = 0.081, *P* = 0.179) (Fig. 3, Panel B). The optimal cut‐offs on the pooled dataset (Youden method) were aCoV = 0.20 (accuracy 85.7%, sensitivity 81.3%, specificity 100%) and ifCoV = 0.24 (accuracy 73.8%, sensitivity 65.6%, specificity 100%). In a binary logistic regression contrasting the pooled PD cohort cases with HC, the overall model was significant (omnibus χ^2^(5) = 29.88, *P* < 0.001), with good explanatory power (Cox–Snell R^2^ = 0.509; Nagelkerke R^2^ = 0.764). The model correctly classified 92.9% of participants. The multivariable analysis showed that only aCoV remained independently associated with PD status (Wald = 4.47, *P* = 0.034; odds ratio ≈1.83 × 10^13^). V showed a nonsignificant trend (Wald = 3.12, *P* = 0.077), whereas Δa, and ifCoV were not significant (all *P* ≥ 0.13).

### Relationship with Clinical Anchors

Total MDS‐UPDRS‐III showed a good and significant correlation with disease duration (*ρ* = 0.362, *P* = 0.021, *q* < 0.05), while item 3.4 alone did not (*ρ* = 0.184, *P* = 0.157 *q* > 0.05). Spearman's rank correlation found that MDS‐UPDRS‐III correlated positively with aCoV (*ρ* = 0.547, *P* = 0.001, *q* < 0.05) and Δa (*ρ* = 0.414, *P* = 0.009, *q* < 0.05), and negatively with V (*ρ* = −0.34, *P* = 0.05, *q* > 0.05). The relationship between ifCoV and MDS‐UPDRS‐III was not significant (*P* = 0.086, *q* > 0.05).

For item 3.4, significant positive associations were observed for aCoV (ρ = 0.387, *P* = 0.014, *q* < 0.05), and ifCoV (ρ = 0.304, *P* = 0.045, *q* < 0.05), whereas V and Δa were not significant (*P* ≥ 0.37).

**Figure 4:**
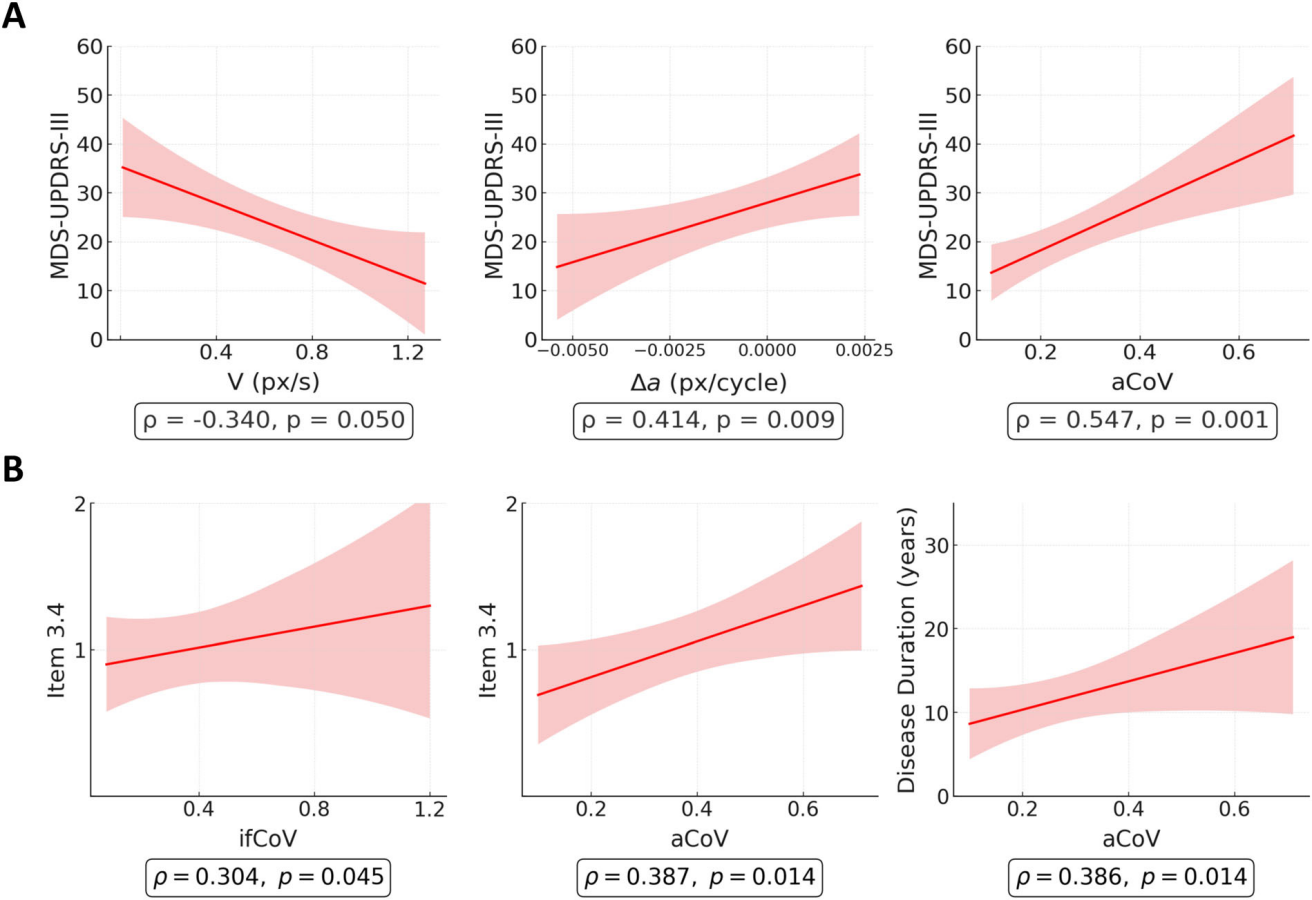
Relationship between finger‐tapping kinematic features and clinical measures in the pooled PD cohort. Δa, amplitude decrement; aCoV, amplitude coefficient of variation; MDS‐UPDRS‐III, Movement Disorder Society‐Unified Parkinson's Disease Rating Scale; PD, Parkinson's disease; px, pixels; V, Velocity.

Regarding disease duration, a positive association emerged for aCoV (*ρ* = 0.386, *P* = 0.014, *q* < 0.05) (Fig. 4), while all correlation tests between the other tapping features and disease duration were non‐significant (*P* ≥ 0.21).

### Relationship with DAT‐SPECT Measures

In the pooled PD cohort, contralateral striatal DAT‐SPECT signal (SBR) showed no significant associations with finger‐tapping kinematics (velocity, amplitude decrement, amplitude variability, or rhythm variability) across striatum, putamen, or caudate (all *P* > 0.05). Trends toward inverse correlations between SBR and global motor severity did not reach significance (MDS‐UPDRS‐III: ρ ≈ −0.33 to −0.40 across ROIs), and SBR was likewise unrelated to item 3.4 or disease duration with *P* > 0.05 for all comparisons (Table S3).

## Discussion

In this retrospective, outpatient clinic–video analysis study anchored to DAT‐SPECT, we report three main insights that refine how markerless finger‐tapping kinematics can augment the neurological examination. CV‐based approaches can capture granular components of motor output, including subtle fluctuations and feature variability, providing sensitive and objective metrics for assessing movement patterns and their changes over time.^36^, ^37^, ^38^

First, in this cohort, variability‐based metrics—especially amplitude variability (AUC 0.93)—were consistently the most informative for distinguishing PD from controls. While traditional scales often emphasize decremental metrics of motor performance, increasing evidence suggests that motor variability may also deteriorate as PD progresses, reflecting underlying deficits in motor control and dopaminergic regulation that are not captured by decremental measures of speed or amplitude alone.^33^, ^37^, ^38^, ^39^ However, all patients were evaluated in the ON state, which likely attenuated case–control differences in tapping speed. Speed is highly levodopa‐responsive,^40^ whereas variability subcomponents of finger tapping show limited responsiveness and smaller effect sizes,^32^, ^36^, ^37^ contributing to the superior discriminative power of variability features in our cohort.

Second, we found that tapping features correlated with disease burden in clinically meaningful ways. Amplitude variability was a good measure of global motor severity and disease duration, while amplitude decrement showed a more modest association and tapping velocity trended inversely with severity (MDS‐UPDRS‐III). Variability‐based features (rhythm and amplitude) displayed good correlation also with item 3.4 rating. On the counterpoint, the sequence effect did not correlate with disease duration in this cohort, underscoring that the construct of bradykinesia remains multidimensional, and that clinical descriptors like amplitude reduction and hesitations can therefore conflate kinematic features that are only partially overlapping.^39^ In multivariable classification, only amplitude variability remained independently associated with PD status, outperforming amplitude decrement (AUC 0.38). Additionally, unlike MDS‐UPDRS‐III, which requires a time‐consuming assessment, these features relied solely on finger tapping videos of the most affected hand in the outpatient setting, reducing the complexity, time resource, and subjectivity associated with traditional assessments.^41^ This simplicity opens new avenues for remote monitoring, particularly in the context of telemedicine, where quick and reproducible measures are essential and counteracting the limitations of shrinking medical resources.^20^, ^41^, ^42^, ^43^, ^44^ Because the metric set is derived from a single, ≤15‐s task with the most‐affected hand, deployment in outpatient and telemedicine settings is logistically simple and rater‐independent, facilitating high‐frequency assessments without additional hardware.

Third, our results contextualize the limited utility of DAT‐SPECT as a progression or severity biomarker at the single‐patient level. In our sample, SBR did not meaningfully couple with the CV‐derived tapping metrics or with clinical scores, reinforcing the principle that imaging evidence of nigrostriatal denervation is valuable diagnostically but insufficient as a universal yardstick for motor severity or kinematic regularity. While dopaminergic loss explains variance in bradykinesia and rigidity, it incompletely accounts for tremor and postural instability.^45^, ^46^ Moreover, early depletion of the more affected putamen may result in a floor effect, reducing the sensitivity to track further progression.^46^ Denervation patterns may significantly vary among individuals, thus making complex the interpretation of SBR as a standalone marker of disease severity.^47^, ^48^

Methodologically, the study flow—screening 148 candidates to a final pooled cohort of 32 PD through systematic quality and timing criteria—favored internal validity at the cost of sample size and generalizability, as confirmed by the wide confidence intervals. As such, results require further external validation, since no training/test split or cross‐validation was used, and classification performance may be subject to overfitting. A key strength is the DAT‐SPECT–anchored case definition: we included only patients with confirmed nigrostriatal denervation, thereby minimizing the risk of enrolling false‐positive PD cases inherent to PD clinical diagnosis.^2^, ^3^, ^7^ Moreover, limiting inclusion to DAT‐anchored PD patients with DAT‐SPECT performed within one year of video acquisition enhanced cohort homogeneity; notably, no left–right differences in SBR were detected, including after adjustment for disease duration. Despite large statistical effect size, heterogeneity of videorecording protocols may have introduced variability across data acquisition processes, which we minimized with a standardized videotaping approach, including a fixed armchair‐camera distance, as outlined in previous publications from our center.^24^, ^25^ This is documented by the low percentage of videos discharged due to technical issues (6/148, 4% of total video sample). Additionally, the retrospective nature of the study limited control over patient recruitment, possibly dampening subtle correlations between video‐extracted data and clinical/neuroimaging markers. To mitigate confounding, we compared finger‐tapping features across groups also after explicit age‐ and sex‐matching, which confirmed our results. Nevertheless, residual confounding cannot be excluded due to minor imbalances in the matched cohort. A minor methodological concern is that we analyzed finger tapping only from the dominant hand in HC; however, in line with our previous studies on the topic,^32^, ^33^, ^34^, ^35^ handedness has no substantial impact on finger‐tapping performance in healthy subjects.

In addition, healthy subjects did not undergo DAT‐SPECT imaging, precluding biomarker confirmation of dopaminergic integrity and limiting case–control comparisons. Finally, we did not assess test–retest reliability or inter‐session stability of kinematic features, that are pivotal to define minimal detectable change and responsiveness.

If further validated, these findings have several clinical and translational implications. For diagnosis, brief, standardized finger‐tapping videos even in the outpatient clinic can yield objective, reproducible indices that complement clinician ratings and may be especially informative in the ON state when speed differences are pharmacologically compressed. For monitoring, variability metrics merit prospective evaluation as candidates for test–retest reliability, minimal detectable change, and responsiveness to both dopaminergic and nondopaminergic interventions. For clinical trials, such metrics could enable high‐frequency, remote outcomes that reduce site burden and capture day‐to‐day fluctuations more sensitively than episodic ratings. And for deployment, their single‐task, single‐hand simplicity favors integration into routine telemedicine, provided that device‐agnostic pipelines, calibration procedures, and quality controls are established. Some studies have already quantified finger tapping in PD with heterogeneous CV approaches, reporting variable performance across pipelines: recent pose‐estimation frameworks (DeepLabCut, MediaPipe, OpenPose) have yielded high AUCs/accuracies in larger datasets (eg, AUC 0.91–0.97), with stage‐aware classifiers showing strong HC vs PD separation but non‐linear behavior of variability metrics across severities.^27^, ^28^, ^49^, ^50^, ^51^ Importantly, substantial between‐study variability in recording conditions (camera geometry, frame rate, lighting), pose‐estimation software, and feature definitions/extraction/usage limits comparability and generalizability, elevating risk of bias.^24^ For example, Heye and colleagues^27^ constructed a motor performance composite score with tapping speed, amplitude and rhythm variability as prevalent features. To support real‐world translation and fair benchmarking of finger‐tapping analytics with CV, shared clinician‐oriented standards for video acquisition and processing are needed (harmonized capture protocols, predefined quality control, and reporting checklists), alongside multisite external validation. Moreover, an open PD dataset with clinically meaningful labels (item scores, disease stage, medication state), coupled with clear governance for privacy, consent, and data sharing, is a major challenge to be addressed.^26^ Although our easy‐to‐use custom CV pipeline yields numerous finger‐tapping features, high‐dimensional video data should augment—not replace—rigorous clinical phenotyping, to avoid overreliance on *big data* at the cost of interpretability.^24^, ^26^, ^52^ In line with previous literature this work confirms the robust accuracy, ease of integration, and adaptability across diverse clinical environments of Mediapipe, highlighting its simpler implementation and lower computational demands compared to other research‐dedicated models.^24^, ^26^ A step forward is a prospective study in asymptomatic or at‐risk individuals, to evaluate whether quantitative CV can detect subclinical motor alterations that precede changes on MDS‐UPDRS‐III or clinician recognition. Such a design would directly test predictive and incremental validity, clarifying whether CV offers earlier and/or more sensitive detection than standard clinical examination.

Looking ahead, we envision three priorities: (i) Prospective validation across ON and OFF states to quantify pharmacodynamic sensitivity of all CV‐derived finger‐tapping features (variability‐based vs decremental ones); (ii) Multisite harmonization with predefined acquisition standards, blinded ratings, and external replication to derive clinically actionable cut‐offs and calibrate decision thresholds against established anchors (eg, MDS‐UPDRS‐III categories, progression strata); (iii) Automation of the entire workflow—from task recognition to frame‐wise segmentation of MDS‐UPDRS‐III items—using neural networks trained on weakly or self‐labeled data.^41^ Such automation would eliminate manual annotation, scale to longitudinal remote monitoring, and facilitate integration with electronic health records.

To conclude, CV technology provides a mechanism to convert visible gross motor signs into quantitative, rater‐independent measures at the point of care suitable for deep phenotyping even in the outpatient setting. Outpatient CV analysis of finger tapping yields scalable, biologically plausible metrics that discriminate PD from healthy subjects and correlate with disease burden thereby motivating prospective, standardized, and demographically diverse studies to translate these signals into clinical decisions.

## Author Roles

(1) Research project: A. Conception, B. Organization, C. Execution; (2) Statistical Analysis: A. Design, B. Execution, C. Review and Critique; (3) Manuscript Preparation: A. Writing of the first draft, B. Review and Critique.

P.M.P.: 1A, 1B, 1C, 2A, 2B, 3A

L.M.: 1A, 1B, 1C, 2A, 3A

A.C.: 1C, 3A

K.R.D.: 1C, 3A

J.A.: 1C, 3A

J.S.: 1C, 3A

J.S.: 1C, 3A

H.H.D.: 1C, 3A

V.D.L.: 1A, 2A, 3B

A.J.E.: 1A, 2A, 2C, 3B

L.d.B.: 1A, 1B, 2A, 2B, 2C, 3B

M.B.: 1A, 1B, 1C, 2A, 2C, 3B

## Disclosures

**Ethical Compliance Statement:** Enrolled participants provided informed consent to videotaping and the retrospective chart evaluation for patients was authorized by the local IRB (#2019–1312). We confirm that we have read the Journal's position on issues involved in ethical publication and affirm that this work is consistent with those guidelines.

**Funding Sources and Conflicts of Interest:** This research received no external funding. PMP has no disclosures. LM has received honoraria from the International Association of Parkinsonism and Related Disorders (IAPRD) Society for social media and web support, and personal compensation as a consultant/scientific advisory board member for Acadia. LM has received a grant (collaborative research agreement) from the International Parkinson and Movement Disorders Society for the MDS‐UTRS Validation Program (Role: PI), Non‐Profit. AC has no disclosures. KRD has no disclosures. JA has no disclosures. JS has no disclosures. JS has no disclosures. HHD has no disclosures. LW has no disclosures. VDL has no disclosures. AJE has received grant support from the NIH and the Michael J. Fox Foundation; personal compensation as a consultant/scientific advisory board member for Mitsubishi Tanabe Pharma America (formerly, Neuroderm), Amneal, Acorda, Abbvie, Bial, Supernus (formerly, USWorldMeds), NeuroDiagnostics, Inc. (SYNAPS Dx), Intrance Medical Systems, Inc., Merz, Praxis Precision Medicines, Citrus Health, and Herantis Pharma; Data Safety Monitoring Board (chair) of AskBio; and publishing royalties from Lippincott Williams & Wilkins, Cambridge University Press, and Springer. He is co‐inventor of the patent “Compositions and methods for treatment and/or prophylaxis of proteinopathies” with which he cofounded REGAIN Therapeutics to fund preclinical studies. He has no financial relationship with the company and has relinquished the right to any personal income from future treatments. AJE owns no stock in any pharmaceutical company with which he has an advisory relationship. LdB is the scientific director and one of the shareholders of Brain Innovations Srl, a University spinoff of the Campus Bio‐Medico University of Rome. MB has no disclosures.

**Financial Disclosures and Conflicts of Interest:** The authors declare that there are no additional disclosures to report.

## Supporting information


**TABLE S1.** Shapiro–Wilk Normality Tests for Clinical, DAT‐SPECT, and Kinematic Measures. Null hypothesis: Gaussian distribution. *P* < 0.05 indicates a significant departure from normality.

**TABLE S2.** Within‐Group Right–Left Hemisphere Effects on log(SBR) by ROI in the pooled cohort mixed‐effects models adjusted for disease duration).

**TABLE S3.** Spearman correlations between contralateral striatal SBR and kinematic and clinical measures.

## Data Availability

The data that support the findings of this study are available from the corresponding author upon reasonable request.
