# Supplementary material for: Computer Vision Analysis for Objective Motor Assessment in Parkinson's Disease: A Retrospective Study
Source: Mov Disord Clin Pract. 2025 Dec 20;13(5):1266–76. doi: 10.1002/mdc3.70488 (PMC13172757; doi:10.1002/mdc3.70488)
Supplement: Supplementary file 1 — TABLE S1. Shapiro–Wilk Normality Tests for Clinical, DAT‐SPECT, and Kinematic Measures. Null hypothesis: Gaussian distribution. P < 0.05 indicates a significant departure from normality. [file MDC3-13-1266-s001.docx]

**Table S1**. Shapiro–Wilk Normality Tests for Clinical, DAT-SPECT, and Kinematic Measures. Null hypothesis: Gaussian distribution. *p*<0.05 indicates a significant departure from normality.

| Group | Variable | Category | Shapiro–Wilk (*W*) | *p*-value | Normality (interpretation) |
| --- | --- | --- | --- | --- | --- |
| Clinical | Age at onset | Clinical | 0.968 | > 0.05 | Not rejected (Gaussian) |
|  | MoCA score | Clinical | 0.913 | 0.013 | Rejected (non-normal) |
|  | MDS-UPDRS-III | Clinical | 0.923 | 0.026 | Rejected (non-normal) |
|  | Age at video | Clinical | 0.944 | 0.096 | Not rejected (Gaussian) |
|  | LEDD | Clinical | 0.716 | <0.001 | Rejected (non-normal) |
|  | Hoehn–Yahr stage | Clinical | 0.776 | <0.001 | Rejected (non-normal) |
|  | Disease duration | Clinical | 0.732 | <0.001 | Rejected (non-normal) |
| SBR | Striatum | DAT-SPECT | 0.951 | 0.015 | Rejected (non-normal) |
|  | Caudate | DAT-SPECT | 0.905 | <0.001 | Rejected (non-normal) |
|  | Putamen | DAT-SPECT | 0.294 | <0.001 | Rejected (non-normal) |
|  | Putamen-to-Caudate ratio | DAT-SPECT | 0.595 | <0.001 | Rejected (non-normal) |
| CV tapping features | Δa (px/cycle) | Kinematic | 0.9773 | 0.5609 | Not rejected (Gaussian) |
|  | V (px/s) | Kinematic | 0.3176 | <0.001 | Rejected (non-normal) |
|  | aCoV | Kinematic | 0.9068 | 0.0023 | Rejected (non-normal) |
|  | ifCoV | Kinematic | 0.7159 | <0.001 | Rejected (non-normal) |

**Abbreviations:** SBR= Striatal Binding Ratio; LEDD = Levodopa Equivalent Daily Dose; CV= Computer Vision; V= Velocity; Δa= amplitude decrement; aCoV= amplitude coefficient of variation; ifCoV= instantaneous frequency coefficient of variation; HC= healthy controls; PD= Parkinson’s disease; MDS-UPDRS-III= Movement Disorder Society-Unified Parkinson’s Disease Rating Scale; px= pixels
