# Supplementary material for: Computer Vision Analysis for Objective Motor Assessment in Parkinson's Disease: A Retrospective Study
Source: Mov Disord Clin Pract. 2025 Dec 20;13(5):1266–76. doi: 10.1002/mdc3.70488 (PMC13172757; doi:10.1002/mdc3.70488)
Supplement: Supplementary file 2 — TABLE S2. Within‐Group Right–Left Hemisphere Effects on log(SBR) by ROI in the pooled cohort mixed‐effects models adjusted for disease duration). [file MDC3-13-1266-s003.docx]

**Table S2.** Within-Group Right–Left Hemisphere Effects on log(SBR) by ROI in the pooled cohort mixed-effects models adjusted for disease duration).

| **ROI** | **Right−Left β [95% CI]** | ***p*-value** |
| --- | --- | --- |
| Striatum | −0.360 [−1.070, 0.350] | 0.3203 |
| Putamen | 0.440 [−0.420, 1.300] | 0.3160 |
| Caudate | 0.460 [−0.370, 1.290] | 0.2774 |
| Putamen/Caudate | −0.160 [−0.470, 0.140] | 0.2959 |

Abbreviations= ROI= Region of Interest; CI= Confidence interval.
