# Supplementary material for: Computer Vision Analysis for Objective Motor Assessment in Parkinson's Disease: A Retrospective Study
Source: Mov Disord Clin Pract. 2025 Dec 20;13(5):1266–76. doi: 10.1002/mdc3.70488 (PMC13172757; doi:10.1002/mdc3.70488)
Supplement: Supplementary file 3 — TABLE S3. Spearman correlations between contralateral striatal SBR and kinematic and clinical measures. [file MDC3-13-1266-s002.docx]

**Table S3.** Spearman correlations between contralateral striatal SBR and kinematic and clinical measures.

| Group | ROI | Target | *ρ* | *p*-value |
| --- | --- | --- | --- | --- |
| Pooled PD | Striatum | V | 0.021 | 0.9083 |
|  |  | Δa | -0.115 | 0.5308 |
|  |  | ifCoV | -0.067 | 0.7169 |
|  |  | aCoV | -0.183 | 0.3155 |
|  |  | MDS-UPDRS-III | -0.382 | 0.0711 |
|  |  | Item 3.4 | -0.014 | 0.9401 |
|  |  | Disease duration | -0.148 | 0.4179 |
|  | Putamen | V | 0.056 | 0.7595 |
|  |  | Δa | -0.153 | 0.4023 |
|  |  | ifCoV | -0.008 | 0.9666 |
|  |  | aCoV | -0.150 | 0.4125 |
|  |  | MDS-UPDRS-III | -0.395 | 0.0954 |
|  |  | Item 3.4 | 0.072 | 0.6959 |
|  |  | Disease duration | -0.097 | 0.5986 |
|  | Caudate | V | 0.084 | 0.6468 |
|  |  | Δa | -0.096 | 0.5995 |
|  |  | ifCoV | -0.129 | 0.4813 |
|  |  | aCoV | -0.148 | 0.4195 |
|  |  | MDS-UPDRS-III | -0.331 | 0.0839 |
|  |  | Item 3.4 | -0.055 | 0.7638 |
|  |  | Disease duration | -0.034 | 0.8544 |

**Abbreviations:** ROI= Region of Interest; V= Velocity; Δa= amplitude dcerement; aCoV= amplitude coefficient of variation; ifCoV= instantaneous frequency coefficient of variation; HC= healthy controls; PD= Parkinson’s disease; MDS-UPDRS-III= Movement Disorder Society-Unified Parkinson’s Disease Rating Scale
